# Supplementary material for: Incorporation of iPSCs together with TERT-immortalized keratinocytes and fibroblasts into reconstructed human gingiva enhances phenotype of gingival epithelium
Source: PLoS One. 2025 Jul 21;20(7):e0327728. doi: 10.1371/journal.pone.0327728 (PMC12279103; doi:10.1371/journal.pone.0327728)
Supplement: Attachment — Submitted filename: Response to Reviewers.docx [file pone.0327728.s002.docx]

June 05, 2025

Response Letter Research Article, Manuscript Number: PONE-D-25-15760

Dear Editor,

We would like to thank you and the reviewers for providing valuable comments and for their time and effort reviewing our work. These reviewer comments combined have provided constructive and helpful criticism of our work and allowed us to further improve the quality of our manuscript.

We have clearly identified the responses in the letter and kept the track changes on in the manuscript. As requested we also provide an unmarked version of the revised manuscript. Please find the response to reviewer questions below and revised manuscript.

We appreciate your consideration of our manuscript for publication in PLOS ONE.

With Kind Regards,

Sue Gibbs

When submitting your revision, we need you to address these additional requirements.

1. Please ensure that your manuscript meets PLOS ONE's style requirements, including those for file naming. The PLOS ONE style templates can be found at

https://journals.plos.org/plosone/s/file?id=wjVg/PLOSOne_formatting_sample_main_body.pdf and

<https://journals.plos.org/plosone/s/file?id=ba62/PLOSOne_formatting_sample_title_authors_affiliations.pdf>

Answer: The style requirements have been checked and the manuscript meets the style requirements.

2. When completing the data availability statement of the submission form, you indicated that you will make your data available on acceptance. We strongly recommend all authors decide on a data sharing plan before acceptance, as the process can be lengthy and hold up publication timelines. Please note that, though access restrictions are acceptable now, your entire data will need to be made freely accessible if your manuscript is accepted for publication. This policy applies to all data except where public deposition would breach compliance with the protocol approved by your research ethics board. If you are unable to adhere to our open data policy, please kindly revise your statement to explain your reasoning and we will seek the editor's input on an exemption. Please be assured that, once you have provided your new statement, the assessment of your exemption will not hold up the peer review process.

Answer: All authors agree with making data available on Mendeley Data. The data has been placed in a repository, which will be made public upon acceptance of our manuscript.

3. PLOS requires an ORCID iD for the corresponding author in Editorial Manager on papers submitted after December 6th, 2016. Please ensure that you have an ORCID iD and that it is validated in Editorial Manager. To do this, go to ‘Update my Information’ (in the upper left-hand corner of the main menu), and click on the Fetch/Validate link next to the ORCID field. This will take you to the ORCID site and allow you to create a new iD or authenticate a pre-existing iD in Editorial Manager.

Answer: The corresponding author ORCID ID has been added.

4. We note that you have included the phrase “data not shown” in your manuscript. Unfortunately, this does not meet our data sharing requirements. PLOS does not permit references to inaccessible data. We require that authors provide all relevant data within the paper, Supporting Information files, or in an acceptable, public repository. Please add a citation to support this phrase or upload the data that corresponds with these findings to a stable repository (such as Figshare or Dryad) and provide and URLs, DOIs, or accession numbers that may be used to access these data. Or, if the data are not a core part of the research being presented in your study, we ask that you remove the phrase that refers to these data.

Answer: We have removed the section referring to the data not shown, as it is no a core part of the research presented. See revised text: Page 18, line 429-433.

5. Please review your reference list to ensure that it is complete and correct. If you have cited papers that have been retracted, please include the rationale for doing so in the manuscript text, or remove these references and replace them with relevant current references. Any changes to the reference list should be mentioned in the rebuttal letter that accompanies your revised manuscript. If you need to cite a retracted article, indicate the article’s retracted status in the References list and also include a citation and full reference for the retraction notice.

Answer: The reference list has been reviewed for completeness and correctness. No retracted papers are included in the reference list.

**Review Comments to the Author**

**Reviewer #1**

**Comment 1:**

Referring to line 82, mentioning the 3Rs could further enhance the scientific soundness of the statement: [https://nc3rs.org.uk/who-we-are/3rs](https://eur04.safelinks.protection.outlook.com/?url=https%3A%2F%2Fnc3rs.org.uk%2Fwho-we-are%2F3rs&data=05%7C02%7Cl.bruske%40amsterdamumc.nl%7C3eeec2e541d3415c0c2b08dd872150ac%7C68dfab1a11bb4cc6beb528d756984fb6%7C0%7C0%7C638815301250598648%7CUnknown%7CTWFpbGZsb3d8eyJFbXB0eU1hcGkiOnRydWUsIlYiOiIwLjAuMDAwMCIsIlAiOiJXaW4zMiIsIkFOIjoiTWFpbCIsIldUIjoyfQ%3D%3D%7C0%7C%7C%7C&sdata=sXJh0g6Gz9sJb5QosCcMHB38ewDexUB2Iy%2Fckvmbl4k%3D&reserved=0). Additionally, authors could mention the FDA Modernization Act 2.0.

Answer: See revised introduction: Page 4, line 85-89, “To overcome these limitations …”.

**Comment 2:**
The authors should mention the terms microphysiological system and state organ-on-chip models. (line 86)

Answer: See revised introduction: Page 4, line 90-96, “This has boosted …”.

**Comment 3:**
Referring to lines 89 to 115, the authors must focus on the main thesis statement and limit the text related to organoids.

Answer: See revised introduction: Page 5.

**Comment 4:**
Referring to lines 123 to 126, the authors are advised to compare the models they mentioned. It would help the reader better understand the current model's novelty and limitations.

Answer: See revised introduction: Page 6, line 136-145, “Similar models have been developed …”.

**Comment 5:**
The current research topic is interesting; authors should also consider providing a graphical abstract of their study.

Answer: Thank you for the suggestion. We have added a graphical abstract.

**Comment 6:**
Authors must consistently use the same abbreviation of induced pluripotent stem cells throughout the manuscript.

Answer: The manuscript has been revised and the abbreviations have been updated.

**Comment 7:**
iPSCs are typically cultured on matrigel-coated surfaces; why do authors prefer Lamaninin over matrigel?

Answer: The justification for the use of laminin as coating solution has been added to the subsection “iPSCs” of the Materials and Methods section. See revised materials & methods section: Page 8, line 180-181, “Laminin 521 is an animal-free product.”.

**Comment 8:**
The authors should mention their study's limitations in the discussion section and expand the section on its potential implications.

Answer: See revised discussion: Page 24, line 581-585, “Limitations of this study include …”.

**Comment 9:**
Authors must label all the histological images with the proper cell type/layer.

Answer: The figures and legends have been revised. We added labels for the epithelium and lamina propria to all figures containing histological images, as well as labels for cell type to the figure 5 to make it easier to distinguish between fibroblasts and keratinocytes.

**Reviewer #2**

**Comment 1:**

The study of Brueske et al, aimed to determine whether incorporation of the human iPSCs into the hydrogel of the human TERT-immortalized keratinocytes and fibroblasts would improve the phenotype of the reconstructed gingival epithelium. Interestingly that such reconstructed human gingiva models demonstrated augmented keratinocyte proliferation within the epithelium, enhanced the thickness of the resultant epithelium, improved differentiation and interdigitation of the epithelium into the collagen hydrogel compared to the standard model developed from TERT cells only. Based on these data the authors conclude that the new iPSC-supplemented gingival model phenotypically resembles the native gingiva much accurately than the earlier models. The study characterises the models with examining morphological changes, i.e.: H&E histology and immunohistochemistry chromosome in situ hybridization and the TUNEL assay. Overall, the data look convincing, however, it is highly recommended that in the future studies the authors will employ combined assessment of structural and physiological changes, for the bilateral characterisation of the models and matching of the form and function.

Answer: We thank the reviewer for these suggestions and agree that future work should look into functionality of the model enhanced with the iPSCs, as well as look into the mechanisms underlying the effect the iPSCs have on the surrounding cells. This has now been added as a future perspective.

See revised discussion: Page 25, line 589-591, “Furthermore, it should be determined…”.

**Comment 2:**
The images with the histology are of very low quality. Dear authors, please provide the images of a publication quality.

Answer: In the PDF build for submission the figures are shown in lower resolution and the higher resolution images can be accessed by clicking on the link in the upper corner. We have also updated the figures with even higher resolution images.

**Comment 3:**
The figure legends are provided inside the chapters of the Results section, which is a bit unusual and inconvenient. Dear authors, please place the figure legends separately, after the References, or after the Figures.

Answer: We are following the guidelines for authors from PLOS One, which request the figure captions to be placed in the manuscript text in read order, immediately following the paragraph where the figure is cited.

**Comment 4:**
In the Figures the P-values are represented as < 0.05, whereas in the text the p-values are given as a certain number, e.g. = 0.0424. Dear authors, please make it uniform for both.

Answer: The manuscript has been updated and the p-values are now mentioned as < 0.05.

**Comment 5:**
In the Figure 6 the images A-D contain the value of the scale bars (i.e. 10um, 100um), whereas the other Figures (5 B, C), 4 (A), 3 (A, B), 2 (A, B), and 1 (A, C) do not have these values. Dear authors, please make things uniform.

Answer: All figures have been updated and the values of the scale bars are now exclusively in the legend of the figures.

**Comment 6:**
In the experiments with the lamina propria (Figure 6) the number of the experiments is not stated. Dear authors, please provide the n-numbers.

Answer: The figured legend has been revised and the number of experiments is now stated. See revised legend: Page 21, line 494-496.

**Comment 7:**
The Latin used throughout the text must be Italic, i.e.: in situ – line 43; e.g. – lines 76, 102, 105, 128; in vitro – line 357, etc.

Answer: The manuscript has been updated and the corresponding text changed to italics.
